# Tannic Acid Tailored-Made Microsystems for Wound Infection

**DOI:** 10.3390/ijms24054826

**Published:** 2023-03-02

**Authors:** Inês Guimarães, Raquel Costa, Sara Madureira, Sandra Borges, Ana L. Oliveira, Manuela Pintado, Sara Baptista-Silva

**Affiliations:** Universidade Católica Portuguesa, CBQF—Centro de Biotecnologia e Química Fina—Laboratório Associado, Escola Superior de Biotecnologia, Rua Diogo Botelho 1327, 4169-005 Porto, Portugal

**Keywords:** chitosan microparticles, tannic acid, antimicrobial, wound infection

## Abstract

Difficult-to-treat infections make complex wounds a problem of great clinical and socio-economic impact. Moreover, model therapies of wound care are increasing antibiotic resistance and becoming a critical problem, beyond healing. Therefore, phytochemicals are promising alternatives, with both antimicrobial and antioxidant activities to heal, strike infection, and the inherent microbial resistance. Hereupon, chitosan (CS)-based microparticles (as CM) were designed and developed as carriers of tannic acid (TA). These CMTA were designed to improve TA stability, bioavailability, and delivery in situ. The CMTA were prepared by spray dryer technique and were characterized regarding encapsulation efficiency, kinetic release, and morphology. Antimicrobial potential was evaluated against methicillin-resistant and methicillin-sensitive *Staphylococcus aureus* (MRSA and MSSA), *Staphylococcus epidermidis, Escherichia coli*, *Candida albicans*, and *Pseudomonas aeruginosa* strains, as common wound pathogens, and the agar diffusion inhibition growth zones were tested for antimicrobial profile. Biocompatibility tests were performed using human dermal fibroblasts. CMTA had a satisfactory product yield of ca. 32% and high encapsulation efficiency of ca. 99%. Diameters were lower than 10 μm, and the particles showed a spherical morphology. The developed microsystems were also antimicrobial for representative Gram+, Gram−, and yeast as common wound contaminants. CMTA improved cell viability (ca. 73%) and proliferation (ca. 70%) compared to free TA in solution and even compared to the physical mixture of CS and TA in dermal fibroblasts.

## 1. Introduction

The skin wound healing process embraces a cascade of coordinated events after a skin injury, trauma, or laceration, which is followed by the natural regeneration of the skin’s protective barrier. In complex wounds, the healing process is characterized by a prolonged and sustained inflammatory phase that impairs dermal and epidermal cells from responding to chemical signals [1]. As a result, wounds are prominent to inflammation and, consequently, to oxidative stress that may lead to delayed or difficult-to-heal, infection, amputations, or even lethal septicemia [1,2].

The problem of complex wounds is thus serious, emerging, and global. In a retrospective analysis of Medicare beneficiaries in 2018, it was reported that 8.2 million people had complex wounds with/without infection, and the cost of wound treatment ranged from $28.1 billion to $96.8 billion. These rising health care costs, as well as the difficult-to-treat infections, make complex wounds a problem of great clinical and socio-economic impact [3].

The mechanisms of wound regulation are attributable to several mediators and many types of cells, including platelets, inflammatory cells, fibroblasts, keratinocytes, also cytokines, growth factors, and matrix metalloproteinases [4]. Any impairment of one of the stages of the normal healing process can cause delay or failure of skin repair. Therefore, the use of substances capable of accelerating healing internally and/or externally is as fundamental as necessary [1,5].

Nonetheless, classic therapies of wound care are increasing the antibiotic resistance and becoming a critical problem, beyond healing. Therefore, there is an emerging demand from researchers around the world to explore bio-based compounds with both antimicrobial and antioxidant activities to heal, strike infection, and fight the inherent microbial resistance. In this sense, plants represent a rich source of phytochemicals, which are more easily absorbed than synthetic drugs and are recognized as accessible treatment options that eliminate the restrictions associated with conventional therapies [6]. Additionally phytochemicals also control infection, inflammation, and the inborn oxidative processes [1], which is a vital phenomenon since the excessive free radical production in response to an injury may hamper the healing process by affecting proteins, lipids, and extracellular matrix elements [6].

Tannic acid (chemical formula C_76_H_52_O_46_) is a water-soluble polyphenol with antioxidant, hemostatic, anti-inflammatory, anticarcinogenic, and antimicrobial activities with low cytotoxicity [1,7]. Its promising properties allows it to be a potential efficient alternative to commercial antibiotics, responding to the need to find new options for wound care. Nonetheless, as a polyphenol, TA may have some drawbacks when targeting wound applications, such as low stability, weak bioavailability, light sensitivity, and consequent decreased biological performance at the wound site [8]. To overcome these limitations, polymeric-based systems (i.e., nano/microsystems, nanoemulsions, hydrogels, films) have been developed as carriers of polyphenols for wound healing, improving its stability, controlling the release kinetics, and therefore increasing the performance and effectiveness [9,10]. Chitosan is one of the most used natural polymers as coating materials as well as a common polymer used in microparticulate systems for skin wound dressing [10] and drug delivery [11], due to biocompatibility, biodegradability, antimicrobial activity, and hemostasis capacity [12,13].

Hereupon, it’s intended to design new CMTA, as a promising drug delivery system, capable of controlling oxidation and possible wound infection, thus promoting a faster and effective tissue regeneration.

## 2. Results and Discussion

Chitosan-microparticles-loaded TA were characterized regarding their physicochemical properties, including the size, morphology, FTIR, and DSC analysis. To simulate wound delivery conditions and to predict the behavior of microparticles over a spaced period, until 24 h, a controlled release study of TA encapsulation was performed. Additionally, the product yield of the microencapsulation technique and the association efficiency of microparticles were also assessed. The biological in vitro profile as antioxidant, and antimicrobial systems, as well as the effect upon cell viability and proliferation, were also tested.

### 2.1. Physicochemical Characterization of Chitosan Microparticles Loaded Tannic Acid

#### 2.1.1. Product Yield

Product yield of spray drying was calculated in order to predict the efficiency of the method in the production of CMTA. The product yield obtained for this process was 32%. Although product yield values of spray drying process may be higher than 50%, recent studies reported, for polyphenols encapsulation into CS microparticles by spray drying, values from 29.63% to 57.3% [14,15,16]. According to these works, product yield obtained is considered a satisfactory value for the laboratory scale and for the materials that were used.

Besides the natural loss of final product associated with adherence of powdered microparticles to the cyclone walls, solid losses from small particles suctioned by the vacuum filter, and the inability of the separation devices to collect the smallest particles [16,17], the yield values might also be affected by the type of encapsulating material. During the microencapsulation process, the adherence of CS to the drying chamber wall was observed, probably caused by its natural viscosity. The viscosity of the initial solution should be the lowest possible to allow homogenous pumping of the solution and atomization [18]. In this sense, the CS chosen was the lowest molecular weight instead the medium molecular weight. Still its viscosity might not be the ideal for the spray drying process. Another way to control the physical properties can be related to the feed temperature: the higher the inlet temperature, the lower the viscosity of the solution and, consequently, the better the conditions to increase the yield value. However, higher values of that temperature may be responsible for degradation of some heat-sensitive compounds, like polyphenols (i.e., TA). Therefore, the choice of inlet temperature value was chosen according to the temperature that can be used safely without damaging the compound [19]. Besides the temperature, the pump rate can also significantly affect the yield of spray drying. According to Plamen D. Katsarov et al. [20], the lower the pump rate of CS solution, the higher the yield of the process, since the quicker the solution is sprayed, the more energy is needed to evaporate the solvent from the particles. Therefore, more experiments should be performed in order to increase the product yield of spray drying, regarding the encapsulation of TA, through the use of other inlet and outlet temperatures, the flow rate, and, consequently, the time of the spray drying process.

#### 2.1.2. Particles Characterization: Size Distribution and Morphology

**Figure 1:**
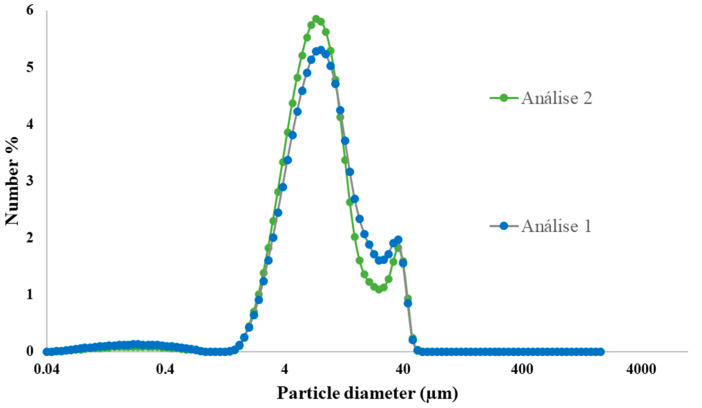
Size distribution in number of CMTA microparticles.

Control of the size and morphology of microparticles is considered an indispensable analysis due to their influence in the sustained and controlled release of encapsulated agents and microparticles stability [21]. Results of size distribution in the number of CMTA microparticles are presented in Figure 1. The results showed that microparticles had a mean diameter of 7.4 μm. This result is concordant to the size of microparticles usually produced by spray drying (1–50 μm) [18,22]. The range of values between 25 μm and 53 μm showed to be discrepant results, probably due to an agglomeration zone of microparticles, as can be observed from the following SEM images (Section 2.3) [23,24]. Therefore, this range was not considered for the calculated mean of the size distribution. Particles with a relative low size value, within the micrometric scale have considerable advantages. In addition, despite the fact that microparticles provide a slower extracellular drug release due to a low surface-to-volume ratio, small sized particles may provide a larger surface area, improve the active compound penetration into wound bed, as well as promote intracellular uptake [25]. Therefore, it is believed that the size of obtained microparticles by spray drying is appropriate for wound care.

#### 2.1.3. Fourier Transform Infrared Spectroscopy

**Figure 2:**
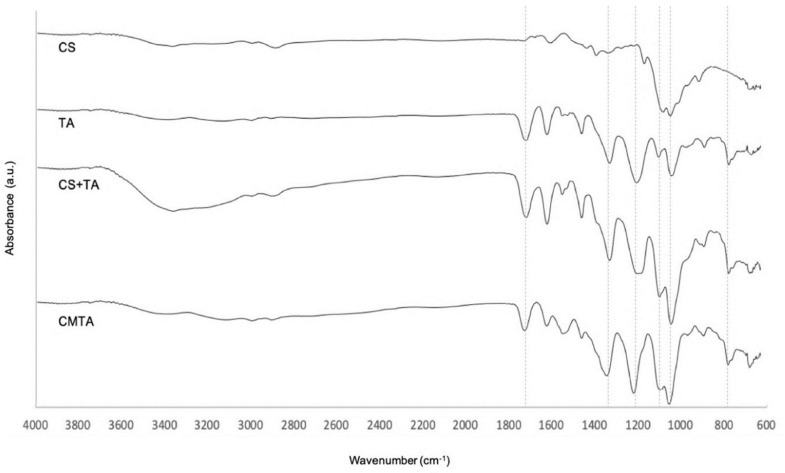
FTIR spectra of CS, TA, CS+TA and CMTA.

Depending on the structure of a compound, its functional groups produce characteristic absorption bands in the spectrum, which are analyzed by FTIR. With these bands, it is possible to draw conclusions about the possible chemical interactions, namely covalent bonds, between compounds. Therefore, FTIR analysis of CMTA, its substrates, and the physical mixture between CS and TA were measured. The results are presented in Figure 2.

Chitosan displays a typical vibrational absorption band between 1595 and 1308 cm^−1^ that are attributed to the stretching of specific bonding of amides. However, the peaks presented on CS spectra are not defined, when compared to reported results. The C–O stretching was identified by the presence of peaks at 1057 and 1021 cm^−1^. At the end, the band located at 896 cm^−1^ is attributed to the stretching of the glycosidic bond [26,27].

Tannic acid spectra were consistent with values given by other studies [28]. Its FTIR spectrum exhibited characteristic bands of aromatic rings in the wavenumber range of 1445–1698 cm^−1^. The two bands around 1314 and 1180 cm^−1^ result from O–H and C–O stretches. The vibration of C=C in benzene rings was identified at 757 cm^−1^ [28].

Spectrum of CMTA microparticles showed that all the above characteristics are maintained at the same wavenumber, indicating no interaction between the drug and carrier. These results are concordant with a previous study [27]. Besides that, no new peaks appeared in the CMTA microencapsulation spectrum or in the physical mixture of the substrate, indicating that no new covalent bonds were detected from the CMTA production. Therefore, the integrity of TA is expected even after the microencapsulation process.

#### 2.1.4. Differential Scanning Calorimetry

**Figure 3:**
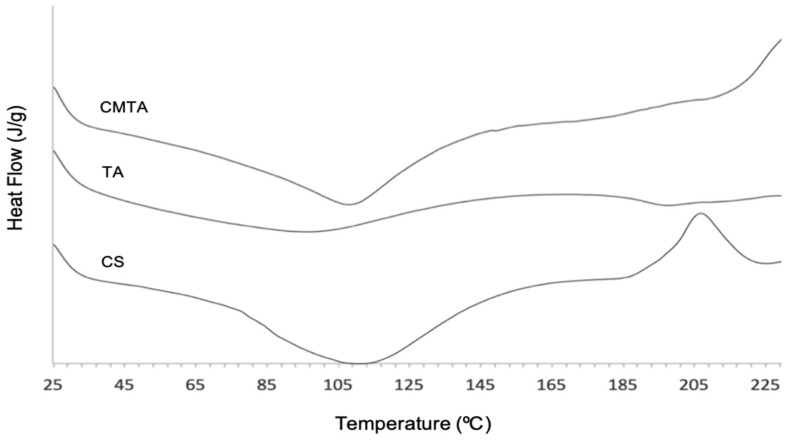
Heat flow vs temperature of CMTA, TA and CS.

Differential scanning calorimetry analyses were performed to provide information about physical and chemical changes of CMTA that involve endothermic and exothermic variations. The graphs of heat flow (J/g), depending on the temperature (°C) of CMTA and its substrates are illustrated in Figure 3.

The results showed a broad endothermic band between 93.69 and 120.67 °C for CS and an exothermic peak 203.20 and 211.70 °C. As other previous studies reported, the endothermic peak, corresponding to a transition that absorbs energy, endorses the loss of water related to hydrophilic groups of CS [29]. Although CS was in powder form, it might have some associated humidity that gave rise to this peak. In turn, the exothermic one, corresponding to a transition that releases energy, is assigned to the thermal degradation of the polymer or melting transition temperature. That degradation may occur due to glycoside bond cleavage or monomer dehydration [29].

Thermogram of TA exhibited a very broad endothermic band at 96.07 °C, related to the evaporation of hydration water molecules, as reported from the literature [30]. Besides that, TA did not show any defined peak, which reveal that the phenolic compound is thermally stable from 25 to 230 °C. At the tested range of temperature, it was not possible to detect the band assessed to thermal degradation, but it was reported that the degradation of this phenolic compound occurs around 260 °C [27].

Chitosan microparticles loaded with TA displayed a sharper endothermic transition on the same values of CS thermogram, due to water that remains after the spray drying process. Its degradation temperature was not detected at tested temperatures, but it can be observed that possibly an exothermic peak would occur above 230 °C. According to Yingju Jing et al. [27], who studied the interaction between TA and CS to functionalize CS, the decomposition peak of TA with CS appeared around 280 °C. Therefore, more assays would be needed at a higher temperature range in order to understand when the degradation occurs. However, based on the obtained results, the interaction between TA and CS led to an increase in the thermal stability of microparticles compared to its substrates.

#### 2.1.5. Association Efficiency

**Figure 4:**
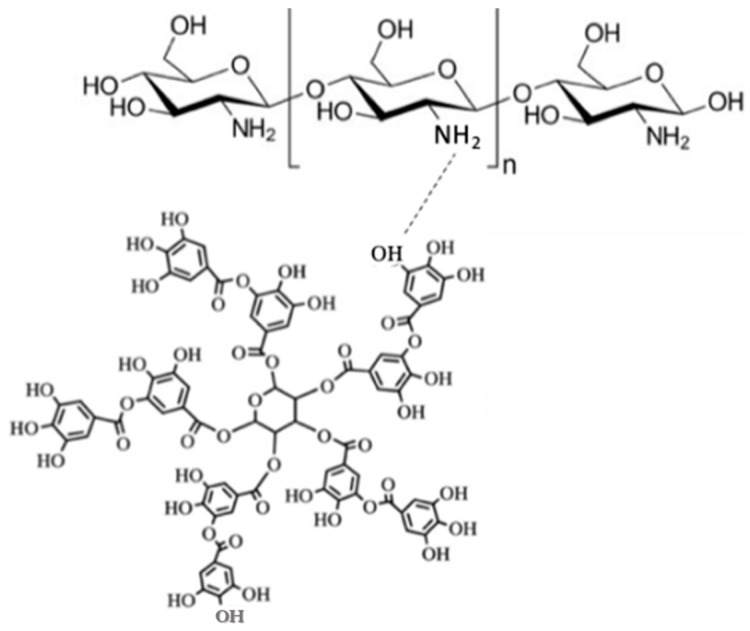
Possible reversible interaction between –OH and –NH3 of TA and CS, respectively.

Tannic acid association efficiency was 98.50 ± 0.02%. Recent studies showed values of AE ranging from 52.7 to 92.6% for polyphenols encapsulated in CS microparticles by spray drying [14,15]. Therefore, AE of the microencapsulation process used in the present work is considered an excellent result when compared to reported values and considering the viscosity of the liquid feed caused by CS. Besides the properties of encapsulating material, pH upon microparticles formation could also have some influence on the AE values for encapsulation of TA in CS microparticles. Chitosan in acidic media can interact with negatively charged groups due to the protonation of chitosan amino groups. Therefore, the polyphenol and the polymer may interact with each other through bonding between hydroxyl groups (–OH) or carboxyl groups (–COO) of TA and hydroxyl (–OH) or amino groups (–NH3) of CS [31,32]. A possible interaction between TA and CS is illustrated in Figure 4. It is believed that there is a higher tendency of interaction with –NH3 because, for CS in acidic media, the positive regions concentrate on the protonated amino group [31,32]. Since that reversible interaction through noncovalent bonds is stronger with lower pH values, it is expectable that the acidic media of feed solution, as well as the low molecular weight of CS, are conditions that may positively influence the amount of TA in CS microparticles produced by spray drying [24].

#### 2.1.6. In Vitro Release of Tannic Acid from Chitosan Microparticles

The controlled release of TA was evaluated in physiological conditions to assure the desirable time and rate in wound bed. Topical delivery conditions were simulated in PBS over 24 h. Results showed a controlled release profile. The release of the core material depends on the type of the encapsulated material, the core-to-coating proportion, as well as the environment where microparticles will be implemented [24].

The peak of TA (6.611 min) was similar to values reported on the study which describes the method used of TA detection [33]. The quantification of the phenolic compound was quick and easily performed.

According to obtained results 0.77 ± 0.002% of the total encapsulated TA was released on the first time point (T0 h), and 0.77 ± 0.003% on the following 24 h (T24 h). The amount of released TA was practically the same (≈0.8%) during the first 24 h.

Results seem to indicate that, probably, the TA content entrapped in the core of the particles was not successfully released in 24 h. Although further testing would have to be done to secure this premise, a possible justification for the obtained values may be related to the amount of TA that can remain on the microparticles surface after the spray drying process [24]. In other words, the amount that was read in HPLC might be the portion of polyphenol in the microparticles’ surface, which means that the microsystem did not even depredate to release TA. The non-degradation of microparticles might be associated with the conditions of the release medium, in particular alkaline pH values of PBS. According to Neculai Aelenei et al. [34], the release of TA is significantly lower in pH values higher than 7.4 such as PBS, due to the partial insolubility of CS in an alkaline medium. However, in acidic medium, more than 90% of the encapsulated TA is released during the first 20 h. The pH values of diabetic wound/chronic wounds are typically alkaline (from 7.2 to 8.9), which hinder the healing process and create a great environment for the growth and multiplication of bacteria [35]. In contrast, lower pH values on the chronic wound surface provide an acidic environment, which helps the wound healing by controlling wound infections [36]. In this sense due to the alkaline pH of complex wounds, the TA release is expected to be slow over time. In a clinical point of view, this lagging release may allow its bioactivity control for 2 to 3 days, until the wound dressing is replaced by medical or nursing services. This may represent a time-regulated regeneration and infection control. Nonetheless, the chronic wound environments are also characterized by containing degradative enzymes, for instance, lysozymes or proteases [37]. These enzymatic phenomena may promote a fast release of TA entrapped within the core of CS particles, due to an erosion of the encapsulating material (CS) [38]. Therefore, and regardless the slow kinetic profile of TA in this assay, it is believed that a fast release could happen in situ, by the enzymatic and inflammatory cascade typical in the wound bed [39]. Once more and to guarantee the veracity of the obtained results, more studies and tests should be done. Further experiments could be done on the studied CS particles to simulate enzyme degradation in PBS, using Protease XIV from *Streptomyces griseus* at a concentration of 3.2 U/mg and temperature (37 °C), according to previous works [40].

### 2.2. In Vitro Biological Potential of Tannic Acid and Chitosan Microparticles Loaded Tannic Acid

#### 2.2.1. Antioxidant Activity Evaluation

The antioxidant activity of TA free in solution and encapsulated into CS microparticles was analyzed using the ABTS radical scavenging assay. In addition, antioxidant activity of CS and CMTA were also evaluated. The results are present in Table 1. All samples were analyzed in 1% (*w/v*).

As a polyphenol and, consequently, a powerful antioxidant compound due to its abundant phenolic hydroxyl groups, the best antioxidant activity results (917.9 ± 33.0 eq. [Trolox] µmol/g) were obtained for TA (1%) free in solution. As expected, CS had a much lower antioxidant activity due to its insufficient H-atom donors (160.0 ± 10.2 eq. [Trolox] µmol/g) [27]. Comparing to TA free in solution, encapsulated TA showed a significant reduction of antioxidant activity (832.2 ± 80.8 eq. [Trolox] µmol/g), certainly caused by the entrapment of the polyphenol into the microparticles. However, the microencapsulation of TA does not eliminate its antioxidant activity, which is concordant with a previous study related to the microencapsulation of polyphenols using CS as a microcarrier [15]. Despite that significant decay, antioxidant activity of CMTA is still high when compared to TA free in solution, probably due to the amount of TA that remained on the microparticles surface after the spray drying process.

#### 2.2.2. Antimicrobial Potency

Agar diffusion method was used to analyze the antimicrobial activity of TA, CMTA, CS, and acetic acid qualitatively against all six studied microorganisms: MRSA, MSSA, *S. epidermidis*, *E. coli*, *P. aeruginosa*, and *C. albicans*. All the results are plotted in Table 2, where the standard deviation was calculated from the triplicates performed for each experiment.

The obtained values demonstrated that TA was able to inhibit all *Staphylococcus* spp. strains (i.e., MRSA, MSSA, and *S. epidermidis)* from the concentration of 2 mg/mL. Furthermore, TA had shown an inhibition zone against *E. coli* and *C. albicans*, also at a concentration of 2 mg/mL. It is possible to observe that, in most cases, the higher the concentration of TA, the higher the diameter of the inhibition zone. However, the phenolic compound was not able to inhibit the growth of *P. aeruginosa*.

There are different mechanisms proposed to justify tannins’ antimicrobial potential, such as changes in the intracellular functions caused by hydrogen binding of tannins to enzymes, what leads to an extracellular enzyme inhibition and unavailability of substrates for digestion [41]. However, it was shown that the primary site of their inhibitory action is the microbial cell membrane through morphological changes of the cell wall by interaction with proteins, which lead to their precipitation and, consequently, an increase of the membrane permeability and microorganism death [41,42]. The different behaviors of polyphenols between Gram+ and Gram- are still a controversial issue. It is known that the cell wall is mainly composed of peptidoglycan [43]. Results from a study from Guofeng Dong et al. [43] revealed that TA can link to peptidoglycan of the cell wall, and it may inhibit the formation of the biofilm. However, a Gram- bacterium has an outer membrane layer composed by lipopolysaccharide molecules and phospholipid that is external to the peptidoglycan cell wall. It was proven that *Staphylococcus* spp. was more susceptible to tannins than *P. aeruginosa* due to the lipopolysaccharide molecules negatively charged on the outer membrane. Therefore, normally, tannins have been more effective against Gram+ bacteria than Gram-, which is concordant with the obtained results [43,44,45].

These are the most prevalence of microorganisms in diabetic foot ulcers. The polyphenol and CS were able to kill both *S. aureus*. Antimicrobial activity of CS against *S. aureus* was already reported. Its main underlying mechanism is related to the linkage of positive charged amino groups (NH_3_^+^) and the negatively charged molecules such as proteins, anionic polysaccharides, and nucleic acids in bacterial membrane, leading to altered membrane permeability with the release of cellular contents, causing cell death. Relatively to encapsulated TA, CMTA also showed inhibitory activities against MRSA and MSSA at 6 mg/mL due to a possible synergy between the of CS with the possible released TA caused by hydration of microparticles. The synergy between antimicrobial activity of CS and antimicrobial activity of certain polyphenols, such as caffeic acid, ferulic acid, and hydroxycinnamic acid were already studied and validated [46]. These results are in accordance with previous studies regarding nanofibrous scaffolds of CS and TA that exhibited excellent antibacterial activity against MSSA and *Escherichia coli* [47]. A hydrogel containing polydopamine/TA/CS/poloxamer hydrogel also showed promising in vitro antibacterial results with bactericidal rates against MSSA and *Escherichia coli* under Near-infrared irradiation (NIR) irradiation of 99.994% and 99.91%, respectively [48].

### 2.3. Biocompatibility of Tannic Acid and Chitosan Microparticles Loaded Tannic Acid in Primary Human Dermal Fibroblasts

**Figure 5:**
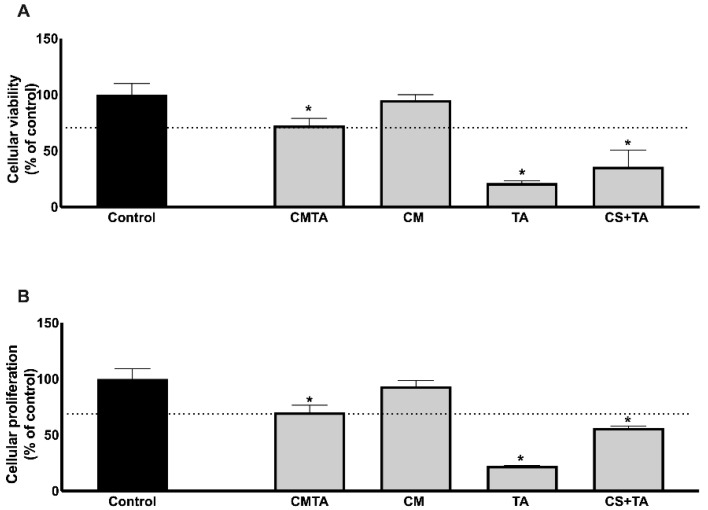
Evaluation of primary human dermal fibroblasts viability by MTT assay (**A**) and proliferation using BrdU incorporation (**B**), after being incubated with TA, CM, CS+TA, CMTA, or with cell culture medium (control group), during 24 h. The data are presented as mean ± SD and are expressed as % of control (n = 3, * *p* < 0.05 versus control group).

In order to evaluate the biocompatibility of CMTA particles, the cytotoxic and proliferative effects were tested in vitro using primary HDF cells. After 24 h of treatment, our results, as depicted in Figure 5, show that free TA presents cytotoxicity (21.39 ± 1.91%) and inhibits the proliferation (22.35 ± 0.55%) of dermal fibroblasts. This effect was minimized once TA is combined with CS solution (CS+TA) and even more reduced in CMTA (72.50 ± 6.51%) for viability assay and (70.08 ± 9.28%) for cell proliferation, when compared to untreated cells (control group).

Since the inhibitory effect is around 30%, it is considered to be clinically acceptable, according to EN ISO 10993-5. The antiproliferative effect of TA is already documented in the literature in vitro and in vivo. Pattarayan and colleagues [49] studied TA, using a mouse embryonic fibroblast cell line and denoted an inhibitory effect on cell viability, in concentrations higher than 10 µM. The authors also described an inhibition on fibroblast proliferation and cell cycle arrest, under tissue growth factor (TGF)-β1 stimulation, pointing out a therapeutic role for TA in preventing pathological fibrosis. Adhesiveness and physiochemical characteristics of an enzymatically crosslinked hydrogel based on chitosan and alginate after TA post-treatment, also revealed significantly high adhesive strength (up to 18 kPa), storage modulus (40 kPa), and antioxidant activity (>96%), antibacterial activity, proliferation, and viability of 3 T3-L1 fibroblast cells [50].

A pre-clinical study [51] described a cardioprotective effect of TA in a mouse-induced model of cardiac fibrosis, possibly through the suppression of toll like receptor 4 (TLR4)-mediated nuclear factor kappa B (NF-κB) signaling pathway.

Fibrosis is a common complication after a skin injury, especially in chronic wounds, suggesting that CMTA could be topically applied as a debridement product, to exert antioxidant effect, control infections and prevent fibrosis at the wound site.

#### 2.3.1. Particles Morphology with Cells

**Figure 6:**
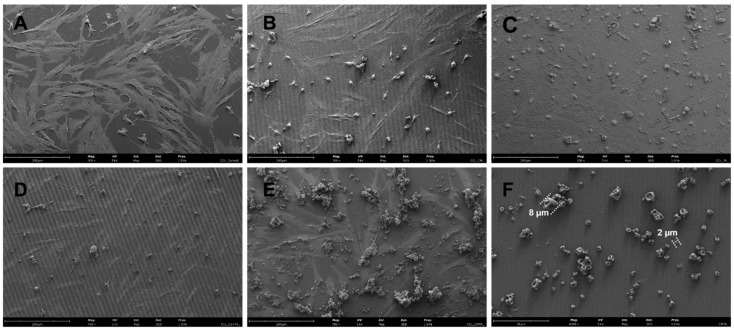
SEM images of using primary HDF cells control (**A**), and HDF cells with: CM (**B**), TA (**C**), physical mixture of CS with TA (**D**), CMTA (**E**), and CMTA control without cells (**F**). Magnification of 700 times for (**A**–**E**), and 4000 times for F sample, with beam intensity 5 kV.

In order to evaluate the effect of CMTA on primary HDF cells morphology, SEM analysis was performed, and the images are presented in Figure 6. CMTA microcapsules (control without cells) were also viewed and showed to be polydisperse, with a particle size ranging between 2 and 10 μm with no relevant differences, which was in accordance with size measurements presented in Section 2.3.1.

The CMTA exhibited a spherical shape with some concavities on the outer surface. The observed tendency to agglomerate was expected due to the microencapsulation process used [24]. According to some studies, the type of morphology obtained for powdered microparticles, also called “raisin-like”, is typical for CS microparticles as well as for CS microparticles-loaded polyphenols produced by spray drying [15,52,53,54]. This roughness and recesses of particles may be caused by the rapid evaporation of drops of liquid during the drying process in the atomizer, and even by the interaction of amino groups of CS (positively charged groups) within the polymer itself [15,55]. Traditionally, the surface of microparticles is normally smooth, but, although there is little information about the impact of the surface on the release efficacy, it is believed that a rough surface with some concavities might be favorable for tissue healing and cell growth due to the similarity of its structure to the extracellular matrix network as well as to a strong surface adhesion [56]. Therefore, regardless how the microparticles would be applied in practice, collapsed, or swelled, their morphology in both cases can be considered appropriated for wound application.

In these SEM micrographs, the beneficial effect of the encapsulation of TA in CS particles can be verified by the adequate adaptability of HDF cells in morphology and proliferation, after contact with CMTA in comparison with both free TA in solution and even with physical mixture with CS. This demonstrates the beneficial protective effect of the particle, as well as its prolonged release profile. The results are in agreement with previously above-mentioned cell culture assays and with other TA reports [49,51].

## 3. Materials and Methods

### 3.1. Standards

All standards and reagents, including TA powder, CS of low molecular weight with a viscosity lower than 100 mPa·s and a deacetylation degree of 85%, acetic acid, 2,2-azinobis (3-ethyl-benzothiazoline-6-sulforic acid) and ethanol (96%) were obtained from Sigma-Aldrich (St. Louis, MO, USA). For HPLC analysis, methanol (100%) was obtained from VWR International (Radnor, PA, USA). Ultrapure water was obtained in the laboratory using Milipore Mili-Q water purification equipment (Millipore, Bedford, MA, USA).

### 3.2. Microbial Strains and Inoculum Preparation for Antimicrobial Experiments

Stock cultures, including methicillin-resistant *Staphylococcus aureus* (MRSA), methicillin-sensitive *Staphylococcus aureus* (MSSA), *Staphylococcus epidermidis, Escherichia coli*, *Candida albicans*, and *Pseudomonas aeruginosa*, were used for antimicrobial activities evaluation of TA free in solution and in CMTA. Test organisms were first activated from glycerol by transfer in nutrient broth at 37 °C for 24 h, then streaking on Mueller–Hinton agar (MHA) (Sigma-Aldrich, USA). A single pure colony was streaked on MHA and incubated at 37 °C for 24 h. Then the concentrations of microorganisms were adjusted with the turbidity of 0.5 McFarland (equal to 1.5 × 10^8^ colony-forming units (CFU)/mL). Turbidity of the microbial suspensions were prepared in sterile saline solution and measured at 600 nm using a mini 1240 UV-Vis spectrophotometer (Shimadzu Corp., Kyoto, Japan), followed by the experiment.

### 3.3. Preparation of Chitosan Microparticles Loaded Tannic Acid

**Figure 7:**
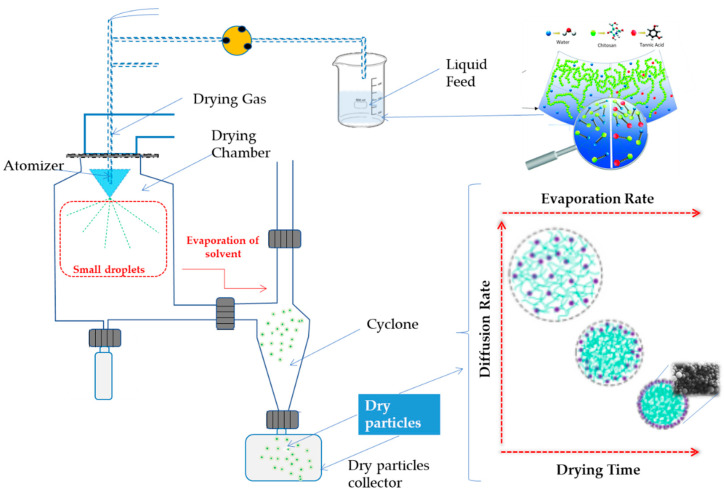
Schematic illustration of CMTA production by spray-dryer.

Chitosan solution was prepared with a concentration of 1% (*w/v*) in an aqueous solution of 1% (*v/v*) acetic acid. Tannic acid solution of 6% (*w/v*) was mixed with CS solution in a proportion of 1:5 (*v/v*). Mixture solutions were prepared with deionized water at room temperature and homogenized, protected from light, for 1 h before the spray-drying procedure. Microencapsulation was performed using a BÜCHI mini spray dryer B-191 (Buchi, Barcelona, Spain). The mixture was fed into the spray dryer under the following conditions: inlet temperature, flow rate, as well as air pressure were, respectively, set at 115 °C, 3.90 mL/min (17%), and 6 bar [15]. The solution was dispersed into fine droplets through a 0.7 mm nozzle. The outlet temperature was kept at 60 °C to preserve the stability of the compounds. The dried powder was collected and stored protected from light in a desiccator. A schematic illustration is presented in Figure 7.

### 3.4. Physicochemical Characterization of Chitosan Microparticles Loaded Tannic Acid

#### 3.4.1. Product Yield

Product yield (%) was calculated for microencapsulation experiment and was expressed as the ratio of the mass of powder collected after drying to the content of the initial infeed solution (Equation (1)).

(1) $$\mathrm{Product}\;\mathrm{yield}\;\left(\%\right)=\frac{\mathrm{Mass}\;\mathrm{of}\;\mathrm{powder}\;\mathrm{obtained}\;\mathrm{at}\;\mathrm{the}\;\mathrm{spray}\;\mathrm{dryer}}{\mathrm{Mass}\;\mathrm{of}\;\mathrm{the}\;\mathrm{initial}\;\mathrm{feed}\;\mathrm{solution}}\times 100$$

#### 3.4.2. Particle Characterization: Size Distribution

Particle size distribution was measured by Coulter-LS 230 particle size analyzer (Beckman Coulter Inc., Miami, FL, USA). Before the analysis, suspensions were prepared in water by adding 0.1% (*w/v*) of powdered microparticles, following by vortexing. The particles were characterized considering a number distribution. Three replicates were performed. Size distribution was expressed in terms of the mean diameter.

#### 3.4.3. Fourier-Transform Infrared Analysis

Fourier-transformed infrared analysis (FTIR) was used to evaluate the structure of TA, CS, and CMTA. The structure is generally interpreted through absorption bands based on the specific vibration of the chemical bonds of each substance. Infrared spectroscopy analysis was performed in a Spectrum 100 FTIR spectrometer equipped with a horizontal attenuated total reflectance sampling accessory (PIKE Technologies, Scientific Products, Pleasantville, NY, USA), the Horizon MBTM FTIR software, and a diamond/ZnSe crystal. All spectra were acquired using 16 scans and a 4 cm^−1^ resolution in the region of 4000–600 cm^−1^. In addition, baseline, point adjustment, and spectra normalization were performed.

#### 3.4.4. Differential Scanning Calorimetry

The thermal analysis of TA, CS, and CMTA were performed using a differential scanning calorimetry—DSC (Shimadzu DSC 60, Scientific Instruments, Columbia, MD, USA). A 5.0 mg portion of each sample was crimped in a standard aluminium pan and heated from 25 to 230 °C at a constant heating rate of 10 °C/min under constant purging of nitrogen at 20 mL/min.

#### 3.4.5. Association Efficiency

Association efficiency (AE) was evaluated considering the amount of TA associated with the microparticles. The AE was measured by the difference between the total TA used to prepare the particles and the amount of residual TA in the solution immediately after dispersion of the particles in water [57]. The AE of TA was obtained according to the following expression (Equation (2)):

(2) $$\mathrm{AE}\;(\%)=\frac{\mathrm{Total}\;\mathrm{amount}\;\mathrm{of}\;\mathrm{TA}-\mathrm{Free}\;\mathrm{amount}\;\mathrm{of}\;\mathrm{TA}\;\mathrm{in}\;\mathrm{supernatant}}{\mathrm{Total}\;\mathrm{amount}\;\mathrm{of}\;\mathrm{TA}\;}\times 100$$

#### 3.4.6. In Vitro Release of Tannic Acid from Chitosan Microparticles

The release of TA from CMTA was tracked to predict the diffusion and kinetic behaviour of the microsystems, and it was tested in simulated physiological environment. For this purpose, 0.1% (*w/v*) of CMTA were suspended in phosphate-buffered saline (PBS), and transferred to clean Eppendorf tubes, followed by placement in a water bath at 37 °C under stirring [57]. PBS was used to simulate physiological conditions at pH 7.4, and its ionic strength was 0.075 M, which is in the optimal range for physiological environment proof-of-concept testing and characterization. Aliquots were collected from the bath over time (0 min, 30 min, 1 h, 2 h, 4 h, 6 h, 8 h, and 24 h) and centrifugated at 14,000 rpm for 5 min (BOECO, Hamburg, Germany). After centrifugation, supernatants were analysed by high performance liquid chromatography (HPLC) to calculate the amount of TA released from the microparticles over the specified time. The quantification was performed by HPLC using the following described method.

#### 3.4.7. High Performance Liquid Chromatography Analysis and Tannic Acid Quantification

Chromatographic analysis was performed using the Waters Alliance e2695 Separate Module HPLC. The results were acquired and processed with Empower^®^ 3 Software 2010 for data acquisition (Mildford, MA, USA), on an Ace^®^ Equivalence 5 C18 column (250 × 4.6 mm i.d.). The conditions of HPLC analysis were applied according to a method already tested and validated for chromatograms determination of standard phenolic compounds, namely TA, the retention time of which was 4.974 min. The mobile phase was composed of two solvents: Solvent A (acetic acid in water (1:25 (*v/v*)) and Solvent B (methanol), at a flow rate of 0.8 mL/min. The injection volume was 20 μL, and the detection wavelength was 280 nm. The gradient program was begun with 100% of Solvent A and was maintained at that concentration for the first 4 min. For the next 6 min, B decreased to 50% and increased to 80% for the next 10 min. At the last two minutes, B reduced to 50% again. Stock standard solutions of TA (10 mg/mL) were prepared and used to construct the calibration curve (R^2^ = 0.9983), composed of six standard concentrations of the phenolic compound: 0.02, 0.05, 0.1, 0.2, 0.3, and 0.5 mg/mL.

### 3.5. In Vitro Biological Potential of Tannic Acid and Chitosan Microparticles Loaded Tannic Acid

#### 3.5.1. Antioxidant Activity Assessment

The ABTS ((2,20-azinobis (3-ethylbenzothiazoline-6-sulfonic acid) diammonium salt) radical scavenging assay was used to estimate the antioxidant capacity of the encapsulated TA in a 96-well microplate. This method is based on the ability of the antioxidant compounds in solution to capture the ABTS∙+ cation, obtained by the reaction between ABTS and potassium persulfate. The working solution was prepared by mixing 10 mL of the stock solution of 7.4 mM ABTS aqueous solution and 10 mL of 2.45 mM potassium persulfate aqueous solution. The mixture was allowed to react for 16 h at room temperature in the dark. The antioxidant potential was measured according to the percentage inhibition, which was between 20 and 80% after 6 min of the reaction between diluted ABTS and the sample [58]. Three sample replicates were performed for each sample: 1% (*w/v*) of TA, CS, and CMTA. The absorbance was measured using a multidetection plate reader at a wavelength of 734 nm (Synergy H1, Winooski, VT, USA). The percentage of inhibition was then estimated using Trolox (6-hydroxy-2,5,7,8-tetramethylchromane-2-caboxylic acid) [25–175 M] as a standard curve. The result was expressed as equivalent concentration of Trolox (in µmol/g), using the calibration curve.

#### 3.5.2. Antimicrobial Potential

Antimicrobial activities of TA, CS, CMTA, and acetic acid were evaluated using a well diffusion method on MHA. The inhibition zones were reported in centimeters (cm). MRSA, MSSA, *S. epidermidis, E. coli*, *C. albicans*, and *P. aeruginosa* were used as references for the antimicrobial assay. Mueller–Hinton agar plates were inoculated with microbial strain under aseptic conditions, and 20 µL of the test samples were placed and incubated at 37 °C for 24 h. After the incubation period, the diameter of the growth inhibition zones was measured and compared.

### 3.6. Cell Culture Experiments

#### 3.6.1. Cells

Primary human dermal fibroblasts (HDF; ATCC - American Type Culture Collection, Manassas, VA, USA) were cultured in Dulbecco’s modified Eagle’s medium (DMEM) supplemented with 10% (*v/v*) FBS (Invitrogen Life Technologies, Paisley, UK) and 1% (*v/v*) penicillin/streptomycin (Invitrogen Life technologies, UK). All experiments were performed between passages 6 and 10. Tannic acid, CS, and the physical mixture between CS+TA, and the CMTA all at 1% (*w/v*), were dissolved in cell culture medium. Controls were performed using cell culture medium. Cells were maintained at 37 °C in a humidified 5% CO_2_ atmosphere.

#### 3.6.2. MTT Viability Assay

HDF (1 × 10^5^ cells/mL) were allowed to grow until 70–90% confluence and then incubated with each treatment for 24 h. Cellular viability was assessed using MTT reagent, according to the instructions provided by the manufacturer. Optical density was measured at 470 nm in a microplate reader (Synergy H1, Winooski, VT, USA). Results are expressed as percentage of control, which was considered to be 100%.

#### 3.6.3. BrdU Proliferation Assay

HDF (1 × 10^5^ cells/mL) were allowed to grow until 70–90% confluence and then incubated with each treatment for 24 h. Cells were incubated with a 5′-bromodeoxyuridine (BrdU) solution and then in situ detection was performed using an in situ detection kit (Roche, Amadora, Portugal), according to manufacturer’s instructions. Optical density was measured at 550 nm in a microplate reader (Synergy H1, Winooski, VT, USA). Results are expressed as percentage of control, which was considered to be 100%.

#### 3.6.4. Particles Morphology and Interaction with Cells

Scanning electron microscopy (SEM) was used to assess the morphology of the HDFs when exposed to the different treatments (CM, TA, CMTA, physical mixture of the two components of CMTA, CS, and TA). Briefly, after 24 h of treatment, all samples were washed twice with PBS 1×, fixed for 30 min at room temperature with a 2.5% glutaraldehyde and washed twice with ultrapure water. Samples were then dehydrated in a graded series of ethanol (from 10% to 100%) and immersed in hexamethyldisilazane (HMDS). After 5 min, samples were dried under a nitrogen airflow, mounted on aluminium stubs with carbon tape, sputter- coated with a gold/palladium alloy, and viewed using Phenom Pro Desktop SEM (Thermo Scientific, Eindhoven, Netherlands). SEM was operated using the secondary electron detector (SED) with an electron beam of 5 kV and magnifications of 700× (cell culture samples) and 4000× (particle morphology assessment).

### 3.7. Statistical Analysis

Every assay was performed at least in three independent experiments. Statistical analysis was performed using IBM^®^SPSS^®^Statistics software, version 20.0. In order to evaluate the differences in antioxidant levels of TA, CS, and CMTA, t-student test was used. Statistical significance of different groups was evaluated by ANOVA followed by the Dunnett post-hoc test. Differences were considered to be significant at a level of *p* < 0.05.

## 4. Conclusions

There is an emerging demand from researchers around the world to explore natural, biodegradable, and ecological solutions as potent alternatives to conventional antibiotics. In this work, CMTA were designed for the first-time loaded TA, by spray-drying without resorting to other harmful reagents. This method represents an automated and scalable system with green and natural reagents. The systems proved to be promising for wound healing and in TA bioavailability protection. CMTA proved to be a sustained release system, with antioxidant, regenerative, and inflammation control properties and with surprising antimicrobial potential. The developed microsystems were a bactericide against Gram+ bacteria, the predominant microorganism present in diabetic wounds and they proved to promote/increase cell viability and proliferation in human dermal fibroblasts when compared with TA free in solution.

However, additional and more extensive studies should be performed in order to get a better characterization of the microparticles, to understand if TA has antimicrobial potential against other microorganisms and to better understand the physiological behavior in situ.

## Figures and Tables

**Table 1:** Antioxidant activity of TA encapsulated into CS microparticles and free in solution.

	ABTS (eq. [Trolox] µmol/g)
TA	917.9 ± 33.0
CS	160.0 ± 10.2
CMTA	832.2 ± 80.8

**Table 2:** Results of inhibition microbial growth zone (cm) produced by different concentration of TA, CMTA, CS (1–10 mg/mL), and acetic acid against MRSA, MSSA, *S. epidermidis*, *E. coli, P. aeruginosa*, and *C. albicans*, by agar diffusion assay.

		Inhibition Bacterial Growth Zone (cm)			
***Staphylococcus aureus*** **(MRSA)**	**Concentration (mg/mL)**	**TA**	**CMTA**	**CS**	**Acetic Acid**
	**10**	1.65 ± 0.10	1.10 ± 0.14	1.40 ± 0.10	-
			
	**8**	1.55 ± 0.10	1.10 ± 0.00	1.3 ± 0.14	-
			
	**6**	1.55 ± 0.10	1.05 ± 0.07	0.80 ± 0.00	-
			
	**4**	1.35 ± 0.10	-	-	-
	
	**2**	0.90 ± 0.00	-	-	-
	
	**1**	-	-	-	-

***Staphylococcus aureus*** **(MSSA)**	**10**	1.75 ± 0.07	1.15 ± 0.10	1.20 ± 0.35	-
			
	**8**	1.40 ± 0.00	1.10 ± 0.00	1.1 ± 0.07	-
			
	**6**	1.45 ± 0.07	0.90 ± 0.00	0.8 ± 0.00	-
			
	**4**	1.45 ± 0.07	-	-	-
	
	**2**	0.90 ± 0.14	-	-	-
	
	**1**	-	-	-	-

***Staphylococcus epidermidis***	**10**	1.45 ± 0.07	1.20 ± 0.28	1.30 ± 0.42	-
			
	**8**	1.40 ± 0.14	0.90 ± 0.00	1.20 ± 0.00	-
			
	**6**	1.40 ± 0.00	0.50 ± 0.01	1.10 ± 0.00	-
			
	**4**	1.15 ± 0.10	-	-	-
	
	**2**	0.85 ± 0.07	-	-	-
	
	**1**	-	-	-	-

***Escherichia coli***	**10**	1.40 ± 0.00	0.80 ± 0.00	1.10 ± 0.14	-
			
	**8**	1.30 ± 0.14	0.65 ± 0.02	1.00 ± 0.10	-
			
	**6**	1.30 ± 0.10	0.60 ± 0.14	1.00 ± 0.10	-
			
	**4**	1.25 ± 0.10	0.60 ± 0.00	0.90 ± 0.02	-
			
	**2**	1.00 ± 0.14	-	-	-
	
	**1**	-	-	-	-

***Candida albicans***	**10**	1.40 ± 0.00	1.00 ± 0.00	1.30 ± 0.00	-
			
	**8**	1.30 ± 0.10	0.80 ± 0.14	1.20 ± 0.14	-
			
	**6**	1.30 ± 0.14	0.75 ± 0.10	1.20 ± 0.10	-
			
	**4**	1.25 ± 0.07	0.75 ± 0.07	1.10 ± 0.00	-
			
	**2**	1.00 ± 0.00	-	-	-
	
	**1**	-	-	-	-
***Pseudomonas aeruginosa***					
-	*no inhibition zones*				

## Data Availability

Not applicable.
